# Supplementary material for: Gamma-Aminobutyric Acid Supplementation Boosts the Phytohormonal Profile in ‘Candidatus Liberibacter asiaticus’-Infected Citrus
Source: Plants (Basel). 2023 Oct 22;12(20):3647. doi: 10.3390/plants12203647 (PMC10609878; doi:10.3390/plants12203647)
Supplement: Supplementary file 1 [file plants-12-03647-s001.zip › plants-2651453-supplementary.pdf]

plants

Supplementary Materials

Gamma-aminobutyric acid supplementation boosts the phytohormonal profile in '*Candidatus* Liberibacter asiaticus'-infected citrus

Yasser Nehela [1, 2] and Nabil Killiny [1]

[1] Department of Plant Pathology, Citrus Research and Education Center, University of Florida, 700 Experiment Station Rd., Lake Alfred, FL 33850; nabilkilliny@ufl.edu (NK), yasser.nehela@agr.tanta.edu.eg (YN)
[2] Department of Agricultural Botany, Faculty of Agriculture, Tanta University, Tanta 31527, Egypt.
* Correspondence: nabilkilliny@ufl.edu

Table S1: Primers used for gene expression analysis of genes involved in salicylates biosynthesis pathway*

Gene	Accession ID		Primer	Amplicon size (bp)
CsCS	XM_006485798.2	F	ACTGGAACACCCATCCATGT	198
		R	GAAATGAAGCCATCCCAGAA	
CsCM1	XM_006482655.2	F	GGGAGCTTGCTTGATTCTTG	196
		R	GTACGCAGCTTGATGTTGGA	
CsCM2	XM_006483415.2	F	GCGAAAAGTCCCCACTAACA	202
		R	CTTCCTCTGGGCTGAATGTC	
CsADT1	XM_006467360.2	F	TTGGTGCCTAACAGATGTGG	196
		R	CCTTGTGAAAGCCAGAGGAC	
CsTAT	XM_006469841.1	F	GTGGAATTTCAGGGCTATCG	201
		R	TCGAGATGGCTATGTGTTGC	
CsAST1	XM_006476023.2	F	GCTTCTCCAAGCATCTGGTC	201
		R	GTTTGAGCGGACTCCATCAT	
CsAST2	XM_006476024.2	F	GGATGTAGTGGCGCAGAGTT	198
		R	CACCACCATCAGCAACAAAC	
CsPAL	XM_006481431.2	F	GTTCTGGCCTGGCTTCTATG	197
		R	ATAAGAGCTGCCATCGAGGA	
CsAAT	NM_001288910.1	F	CTCGTGTTGATGTGCGTCTT	200
		R	TGGCCTCTCCGGAACTATTA	
CsKAT	XM_006489736.1	F	GGGTACAGTTTTGGCACCAG	200
		R	CAGGCACAGTTTCTGAACCA	
CsICS	XM_006476586.2	F	TGCCGGAACAGGGATAGTAG	197
		R	CTTGGTGAACTGCACAATGG	

* The listed genes were assembled based literatures [1–3] and recent available data in national center for biotechnology information website (NCBI, http://www.ncbi.nlm.nih.gov/gene/).

Abbreviations

CsCS: Chorismate synthase
CsCM1: Chorismate mutase 1
CsCM2: Chorismate mutase 2
CsADT1: Arogenate dehydratase/prephenate dehydratase 1, chloroplastic
CsTAT: Tyrosine aminotransferase
CsAST1: Aspartate aminotransferase, cytoplasmic-like
CsAST2: Aspartate aminotransferase, chloroplastic-like
CsPAL: Phenylalanine ammonia-lyase
CsAAT: Alcohol acyl transferase
CsKAT: 3-ketoacyl-CoA thiolase 2
CsICS: Isochorismate synthase

Table S2: Primers used for gene expression analysis of genes involved in auxins biosynthesis pathway*

Gene	Accession ID		Primer	Amplicon size (bp)
CsASA	XM_015532734.1	F	TTGAGCTTCAACCGGAGACT	199
		R	ACTGAAAGTTGCTGCGGACT	
CsASB	XM_006469235.2	F	CAATCGCACAGTTGCTCAGT	199
		R	GCTCAGGGATTGCAGAGAGA	
CsTS	XM_006482255.1	F	GCAGATGGAGGCTGTAGGAG	201
		R	CCTGCGGTATCACCAAAATC	
CsTSA	XM_006470948.2	F	TACGGAAGGAAGCCATCAAG	197
		R	GGAGTGGTGGGACCTGTAAA	
CsTSB	XM_006493882.2	F	GTGTTGCATGGTGCTTTGAG	199
		R	CAGGTCCAACTCCAGGGTAA	
CsTAA2	XM_015529685.1	F	AATGGAACTGGGTTGTCTGG	199
		R	TCATCAGGGATATGGGAAGG	
CsTAA4	XM_006473060.2	F	CGCATCTGGTTGAGTTTGTG	195
		R	TAGAGCCAGCATGACCAGTG	
CsTDC1	XM_006469752.3	F	CGACTTTATGGCCTGGAAAA	206
		R	GGGGTGACAACCTATCCTCA	
CsYUC2	XM_006466708.2	F	CTGGCCCTGTAATTGTTGGT	194
		R	ACGAGGGAATGGCACATAAG	
CsYUC8	XM_006480095.2	F	CTACAGGGTACCGCAGCAAT	202
		R	ATTTCCTTTCCAGCCATGTG	
CsNIT4	XM_006487697.2	F	GTTGCTGTTTCCAGGGATTG	201
		R	ACAGAATCCGGTGTGAGGTC	
CsAO1	XM_006487737.2	F	GGGATCAGACAGGAATCGAA	240
		R	CCGCCCCAAGAAGTATTGTA	

* The listed genes were assembled based literatures [1–3] and recent available data in national center for biotechnology information website (NCBI, http://www.ncbi.nlm.nih.gov/gene/).

Abbreviations:

CsASA: Anthranilate synthase alpha subunit 1
CsASB: Anthranilate synthase beta subunit 2
CsTS: Tryptophan synthase-like
CsTSA: Tryptophan synthase alpha chain, chloroplastic-like
CsTSB: Tryptophan synthase beta chain 1, chloroplastic-like
***CsTAA2*:** Tryptophan aminotransferase-related protein 2-like
***CsTAA4*:** Tryptophan aminotransferase-related protein 4-like
CsTDC1: Aromatic-L-amino-acid decarboxylase-like (aka tryptophan decarboxylase)
CsYUC2: Indole-3-pyruvate monooxygenase YUCCA2
CsYUC8: Indole-3-pyruvate monooxygenase YUCCA8
CsNIT4: Bifunctional nitrilase/nitrile hydratase NIT4A-like
CsAO1: Indole-3-acetaldehyde oxidase-like (aka Acetaldehyde oxidase)

Table S3: Primers used for gene expression analysis of genes involved in abscisic acid biosynthesis pathway*

Gene	Accession ID		Primer	Amplicon size (bp)
CsZEP	XM_006466537.2	F	GCCGTTGTGCTTCTAGGTTC	203
		R	CGATCACCTTAACCCGAAAA	
CsVDE	NM_001288881.1	F	AATCGCATACCAACCTGCTC	197
		R	GGTTTGAAGGCAAGCAACAT	
CsNSY	NM_001288932.1	F	AACGTTGACCAAACCAGACC	199
		R	CTCACTGAGCACCGAAAGTG	
CsNCED	NM_001288935.1	F	ATGGCGGCAGCAACTACTAC	199
		R	CTGCAGGTGATGGAGGGTAT	
CsABA2	NM_001288867.1	F	GCAATCTGCTGGGCAATAGT	203
		R	ATGCCAGTAGCTCCACCTGT	
CsAAO3	XM_006487736.2	F	TTCCTTCAGGGGATTCTGTG	201
		R	CAACCTGTTTGGGGTACCAG	

* The listed genes were assembled based literatures [1–3] and recent available data in national center for biotechnology information website (NCBI, http://www.ncbi.nlm.nih.gov/gene/).

Abbreviations

CsZEP: Zeaxanthin epoxidase
CsVDE: Violaxanthin de-epoxidase
CsNSY: Neoxanthin synthase
CsNCED: 9-*cis*-Epoxycarotenoid dioxygenase 3
CsABA2: Short chain alcohol dehydrogenase
CsAAO3: Abscisic aldehyde oxidase

Table S4: Primers used for gene expression analysis of genes involved in *trans*-jasmonic acid (*t*JA) biosynthesis pathway*

Gene	Accession ID		Primer	Amplicon size (bp)
CsFAD	XM_006480990.2	F	CAGATCCCGCATTACCACTT	195
		R	GGTATCGGTCCCGATTTCTT	
CsLOX	XM_006483993.1	F	TGGCTGTCCAAGACACTCTG	198
		R	CAGCACCACATCTGCCTTTA	
CsAOS	NM_001288906.1	F	GTTTCAGCTCGCTCCGTTAC	199
		R	GAGGTTGTGACACGCTTCCT	
CsAOC	NW_006260521.1	F	GCGAGTGGGAATTACAGCAG	201
		R	TTAACCTGCCCACTCACTCC	
CsAAE7	XM_006488806.2	F	CCACCAGAGGACACAATCCT	201
		R	CTGAGCTTCGAAAGGGAGTG	
CsOPR3	XM_006475468.2	F	TGCCACTTCTCTTGTTGTGG	198
		R	AAATGGTAGCGTCCCTTCCT	
CsACX1	XM_006477083.2	F	ACGGATCATTGAGCTTCGTC	198
		R	CCCAGTGGAGATCCGTAAAA	
CsAIM	XM_006488772.2	F	TAATCCTCGTGACCCGAGAC	203
		R	CTCTGCTGACGTCAAATCCA	
CsKAT	XM_006480138.2	F	ATACCTGCTGCGGTGAAGTC	200
		R	AGGATCAAGCCCCAGTTTCT	

* The listed genes were assembled based literatures [1–3] and recent available data in national center for biotechnology information website (NCBI, http://www.ncbi.nlm.nih.gov/gene/).

Abbreviations

CsFAD: ω-3 Fatty acid desaturase
CsLOX: Lipoxygenase
CsAOS : Allene oxide synthase
CsAOC : Allene oxide cyclase
CsOPR3 : 12-Oxophytodienoate reductase 3
CsAAE7: Acetate/butyrate--CoA ligase AAE7
CsACX1: Acyl-coenzyme A1
CsAIM1: Enoyl-CoA hydratase, mitochondrial-like
CsKAT: 3-Ketoacyl-CoA thiolase , peroxisomal-like

Table S5: Primers used for gene expression analysis of pathogenesis-related proteins

Gene	Function		Primer	Reference
CsPR1	Antifungal and antioomycetic	F	AACTCGCCTCAAGACTACCT	[4]
		R	TGCAACTGTGTCGTTCCATA	
CsPR2	β-1,3-Glucanase	F	TTCCACTGCCATCGAAACTG	[5]
		R	TGTAATCTTGTTTAAATGAGCCTCTTG	
CsPR3 [a]	Chitinase, antifungal	F	TCCGACTACTGTGATCCTAACT	[6]
		R	TCTCCGCACCGTAAGTAATTG	
CsPR4 [b]	Class I, II Chitinases	F	AGCAAATTGGATGGGACTTG	Newly designed
		R	CCATTGGCACACTGATCAAC	
CsPR5	Thaumatin-like protein	F	CACCATTGCCAATAACCCTAATG	[7]
		R	GGGACAGTTACCGTTAAGATCAG	[8]
CsPR15 [c]	Oxalate oxidase	F	TGGTTTGAGCAAAGAGGGTAATA	[6]
		R	AGAGTATTGAGGCCAGGAAATG	
CsSTH-2 [d]	Pathogenesis-related protein STH-2-like	F	TGGAAGCGTTGAACAAATCA	Newly designed
		R	TACAAATGCAACCTCCACCA	
CsWRKY70	Activator of SA-dependent defense	F	CTGTGCTCGGTACTACTGTTAC	[4]
		R	CGGCGATAGTCATCGGAATTA	

[a] Primers were designed based on sequence *orange1.1g026315m* obtained by searching the homologous pathogenesis-related (*PR*) gene *PR-3* sequence within the citrus genome.

[b] Primers were designed based on sequence of pathogenesis-related protein PR-4A (GenBank XM_006487730.3)

[c] Primers were designed based on sequence *30147.m014037* obtained by searching the homologous *PR* gene sequence within the citrus genome.

[d] Primers were designed based on sequence of pathogenesis-related protein STH-2-like (GenBank XM_015533512.1)

Referances

1. Itoh, H.; Izawa, T. A Study of Phytohormone Biosynthetic Gene Expression Using a Circadian Clock-Related Mutant in Rice. *Plant Signal. Behav.* **2011**, *6*, 1932–1936.
2. Lahey, K. a; Yuan, R.; Burns, J.K.; Ueng, P.P.; Timmer, L.W.; Kuang-Ren, C. Induction of Phytohormones and Differential Gene Expression in Citrus Flowers Infected by the Fungus Colletotrichum Acutatum. *Mol. Plant. Microbe. Interact.* **2004**, *17*, 1394–1401, doi:10.1094/MPMI.2004.17.12.1394.
3. Quecini, V.; Torres, G.A.M.M.; de Rosa, V.E.; Gimenes, M.A.; De, J.B.; De, A. V.; Benedito, V.; Targon, M.L.P.N.N.; Cristofani-Yaly, M.; Rosa Jr, V.E. de; et al. In Silico Analysis of Phytohormone Metabolism and Communication Pathways in Citrus Transcriptome. *Genet. Mol. Biol.* **2007**, *30*, 713–733, doi:10.1590/S1415-47572007000500002.
4. Dutt, M.; Barthe, G.; Irey, M.; Grosser, J. Transgenic Citrus Expressing an Arabidopsis NPR1 Gene Exhibit Enhanced Resistance against Huanglongbing (HLB; Citrus Greening). *PLoS One* **2015**, *10*, e0137134, doi:10.1371/journal.pone.0137134.
5. Francis, M.I.; Redondo, A.; Burns, J.K.; Graham, J.H. Soil Application of Imidacloprid and Related SAR-Inducing Compounds Produces Effective and Persistent Control of Citrus Canker. *Eur. J. Plant Pathol.* **2009**, *124*, 283–292, doi:10.1007/s10658-008-9415-x.
6. Hu, J.; Jiang, J.; Wang, N. Control of Citrus Huanglongbing via Trunk Injection of Plant Defense Activators and Antibiotics. *Phytopathology* **2018**, *108*, 186–195, doi:10.1094/PHYTO-05-17-0175-R.
7. Kim, J.-S.; Sagaram, U.S.; Burns, J.K.; Li, J.-L.; Wang, N. Response of Sweet Orange (*Citrus Sinensis*) to ' *Candidatus* Liberibacter Asiaticus' Infection: Microscopy and Microarray Analyses. *Phytopathology* **2009**, *99*, 50–57, doi:10.1094/PHYTO-99-1-0050.
8. Li, J.; Pang, Z.; Trivedi, P.; Zhou, X.; Ying, X.; Jia, H.; Wang, N. "Candidatus Liberibacter Asiaticus" Encodes a Functional Salicylic Acid (SA) Hydroxylase That Degrades SA to Suppress Plant Defenses. *Mol. Plant-Microbe Interact.* **2017**, *30*, 620–630, doi:10.1094/MPMI-12-16-0257-R.
